# Impact of COVID-19 on Movement Disorders Patients in the Outpatient Setting

**DOI:** 10.7759/cureus.107110

**Published:** 2026-04-15

**Authors:** Chase Kingsbury, Shaila Ghanekar, Joshua U Hancock, Colyn White, Yayi Zhao, Yangxin Huang, Oliver Flouty, Theresa A Zesiewicz

**Affiliations:** 1 Department of Neurology, Ataxia Research Center, University of South Florida, Tampa, USA; 2 Department of Biostatistics, University of South Florida, Tampa, USA; 3 Department of Neurosurgery and Brain Repair, University of South Florida, Tampa, USA; 4 Department of Neurology, James A Haley Veteran’s Hospital, Tampa, USA

**Keywords:** covid 19, emotional well being, healthcare delays, movement disorders and tremors, psychological distress

## Abstract

Introduction

The coronavirus disease 2019 (COVID-19) pandemic may have been the only worldwide infectious outbreak that many patients have experienced in their lifetime. The impact of COVID-19 on patients with chronic neurodegenerative disease, including movement disorders, is largely unknown. Since patients with movement disorders rely heavily on inpatient therapies and are closely followed by their medical team, it would be reasonable to see an increase in stress during a period of prolonged isolation. This study aimed to determine the emotional impact, stress, and burden of the COVID-19 pandemic on patients with movement disorders in an academic practice.

Methods

Over four months during the COVID-19 pandemic, from May 2021 to August 2021, patients with movement disorders were screened for eligibility to participate in the study conducted at a single academic center. Patients must be at least 18 years of age, diagnosed with a movement disorder by an academic physician who specializes in movement disorders, and score ≥ 24 on the mini-mental state exam (MMSE). Patients would be excluded if they were not able to speak or read English or scored <24 on the MMSE, indicating cognitive impairment. Study participants were asked to fill out a subjective questionnaire (the COVID-19 and Movement Disorders Questionnaire [CMDQ]) asking whether neurological symptoms, relationships, mental health care, finances, or healthcare delays worsened during the COVID-19 pandemic. Patients were also tested on the Pandemic Emotional Impact Scale (PEIS), a validated instrument designed to measure the emotional impact of a worldwide pandemic on populations within the United States, and the Patient Health Questionnaire (PHQ-4).

Results

The mean PEIS score for the cohort was 32.52 ± 12.61 (range 16-80). Higher (worse) PEIS scores were significantly associated with “personal financial loss” (p=0.0077), “worsening neurological symptoms” (p=0.0006), “strained relationships” (p=0.0021), “friends/family experiencing financial loss” (p=0.0006), “friends/family hospitalized” (p=0.0178), “delay in healthcare” (p < 0.0001), and “masks impacting health” (p = 0.0064). The mean PHQ-4 score was 3.18 (mild distress), and over 30% of the population were positive for symptoms of anxiety.

Conclusion

Although the emotional burden of COVID-19 was low in this cohort, patients were affected by financial burdens, strained relationships, delays in healthcare, and mask-wearing. More research is needed to assess the potential long-term effects of COVID-19 on these individuals.

## Introduction

The coronavirus family comprises enveloped single-stranded RNA viruses that can become human pathogens through zoonotic transmission [1]. SARS-CoV-2 is the virus responsible for the coronavirus disease 2019 (COVID-19) pandemic, which originated in the Hubei province of China and eventually spread across the globe [1]. As of August 2024, there have been over 776 million confirmed cases of COVID-19 and nearly 7 million confirmed deaths attributed to the disease [2,3]. COVID-19 has had a significant impact on public health, the global economy, and daily life around the world.

Of the many subpopulations affected by COVID-19, patients with movement disorders may be at risk for worsening of their disease state. Movement disorders are a group of neurological diseases that can impair motor function, including Parkinson's disease (PD), essential tremor (ET), and spinocerebellar ataxia (SCA), among others [4]. Movement disorder populations are more likely to have other comorbidities that make them particularly susceptible to contracting COVID-19 and experiencing worsened outcomes. While there are many FDA-approved treatments available for movement disorders, such as carbidopa/levodopa and deep brain stimulation, these therapies only target the symptoms and not the underlying cause of the disease [5]. As a result, many patients with movement disorders rely on physical therapy and activity-based rehabilitation to improve their balance, coordination, and overall quality of life. 

The COVID-19 pandemic has presented numerous challenges to individuals living with movement disorders and to the healthcare providers who treat them. Many doctor appointments and physical therapy sessions were canceled or delayed, which had a significant impact on the quality of life in patients with movement disorders since they rely heavily on regular medical care and rehabilitation [6,7]. In a review of the literature, many studies evaluating the impact of COVID-19 on movement disorder populations only include PD cohorts while excluding other disorders like ET and SCA. Furthermore, approximately 85% of PD patients who recovered from COVID-19 experienced long-term negative sequelae of problems, including worsening motor function, increased levodopa requirements, fatigue, brain fog and other cognitive issues, and sleep disturbances [7]. Although prior studies have explored pandemic-related outcomes in PD, the overall psychosocial impact of COVID-19 across diverse movement disorder diagnoses remains incompletely characterized.

The present study was designed to address this gap by evaluating the emotional and practical impact of the COVID-19 pandemic in patients with movement disorders using patient-reported outcome measures. The primary objective was to assess the association between pandemic-related experiences and emotional well-being. Secondary objectives included examining the relationship between psychological distress and perceived pandemic burden and identifying specific pandemic-related stressors associated with worse outcomes. We hypothesized that individuals reporting greater pandemic-related disruptions would demonstrate higher levels of psychological distress and negative emotional impact. 

## Materials and methods

Study design

From May to August 2021, patients in an academic movement disorders practice were recruited for this cross-sectional study to evaluate the effects of the global COVID-19 pandemic on patients with movement disorders, using a combination of internally and externally generated questionnaires. A cross-sectional design was selected to enable a timely assessment of patient experiences during a defined phase of the pandemic. The study was evaluated and approved by the University of South Florida Institutional Review Board (IRB) (Study # 2386).

Patients were contacted either by phone call or in person, with the research visit occurring at their standard follow-up visit for their relevant movement disorder. Each study patient was educated on the protocol, data collection process, purpose of the study, and associated risks before signing an informed consent form (ICF). Adequate time was given to review the ICF and ask questions with an informed researcher before signing the ICF. Upon completion of the ICF, three questionnaires were administered in succession, namely the internally generated COVID-19 and Movement Disorders Questionnaire (CMDQ) (see Appendix), the externally validated Patient Health Questionnaire (PHQ-4), and the Pandemic Emotional Impact Scale (PEIS). The cross-sectional study design required patients to complete one visit total.

Eligibility criteria

Inclusion Criteria

Each study patient was required to be at least 18 years of age and diagnosed with a movement disorder by an academic physician who specializes in movement disorders. Patients were also required to score ≥ 24 on the mini-mental state exam (MMSE), with a score < 24 indicating cognitive impairment.

Exclusion Criteria

Inability to speak or read English was considered exclusionary for this study. Additionally, patients with an MMSE score <24 were excluded from the study. Once enrolled, subjects could rescind their enrollment for any reason. Thus, their data was excluded from the study.

Clinical rating scales and questionnaires

The PHQ-4 is a brief, validated measure of anxiety and depression symptoms [8]. This scale contains two subscales (anxiety and depression), consisting of two items each, producing a sum score quantifying psychological distress [8]. A score of three or greater on either subscale is considered the cutoff point for identifying possible symptoms of clinical anxiety or depression. The initial question, “Over the last two weeks, how often have you been bothered by the following problems?” is applied to four categories. The answers are scored on a four-point scale characterized as “Not at all (0), Several days (1), More than half the days (2), and Nearly every day (3).” Once completed, the total score and both subscale scores are calculated. The total score ranges from 0 to 12, and participants can be stratified into one of four “psychological distress categories” described as “None (0-2), Mild (3-5), Moderate (6-8), and Severe (9-12)” [9,10].

The PEIS is an externally validated rating scale used to assess the effects of pandemics on emotional well-being. Created in response to the COVID-19 pandemic, this scale can be used to measure the effects of future unknown global pandemics on the United States (US) population. This rating scale consists of 16 questions scored on a one-to-five scale, with five being the worst and total scores ranging from 16 to 80. The initial question, “As a result of the COVID-19 pandemic, did you find that you were...” is applied to each of the 16 unique categories affecting daily life. The answers are characterized as “Not at all (1), A little bit (2), Moderately (3), A lot (4), and Extremely (5)” [11].

The CMDQ was an internally generated questionnaire approved for use by waiver by the USF IRB. The 40-question form covers demographic information and relevant topics concerning the impact of COVID-19. A nonexhaustive list includes subjects such as the history of COVID-19 diagnosis, worsened neurological symptoms, healthcare delays, impact of masks on health, strained relationships, lost jobs, financial loss, and mental health decline. The full questionnaire is linked at the bottom of the article with its reference.

Statistics

Descriptive statistics (mean, standard deviation (SD), frequency distribution) were calculated for demographic and clinical variables. Data was collected and processed in-house, and absent or incomplete responses were excluded for that specific question. Associations between these variables and clinical rating scale responses were analyzed using an independent t-test, analysis of variance, and Pearson’s correlation coefficient. Mean PEIS scores were compared with individual topics from the CMDQ to analyze potential associations via independent t-test, analysis of variance, and Pearson’s correlation coefficient. To evaluate the relationship between the PHQ-4 and PEIS severity, Jonckheere-Terpstra tests were performed. Total scales and subscales were assessed for associations. All analyses and sample size calculations were performed using the R statistical package version 4.3 [12]. 

## Results

Demographical data

Of the 134 participants, the mean age was 68.0 ± 10.5 years, consisting of 72 (54%) males and 62 (46%) females. The vast majority of the cohort was Caucasian, 122 (91%), followed by 6 (4.5%) Asian and 3 (2.2%) “not reported" (Table 1). There were 11 (8.2%) participants who reported Hispanic/Latino descent. The cohort consisted of 83 (62%) PD patients, followed by 16 (12%) ET, 11 (8%) dystonia, 7 (5%) ataxia, and 17 (13%) other (Table 2). High school was the highest level of education for 38 (28%) of participants, followed by 35 (26%) college, and 25 (19%) graduate education (Table 3). More than half of the population, 82 (61%) participants, were retired, 98 (73%) were married, 115 (86%) were living with a spouse, caregiver, or partner, and 88 (66%) denied a history of smoking.

**Table 1:** Descriptive statistics for self-identified races

Race	Frequency	Percent
White	122	91.04
Asian	6	4.48
African American	1	0.75
Mixed	2	1.49
Not Reported	3	2.24

**Table 2:** Descriptive statistics for diseases

Disease	Frequency	Percent
Parkinson's disease	83	61.94
Essential tremor	16	11.94
Dystonia	11	8.21
Ataxia	7	5.22
Other	17	12.69

**Table 3:** Descriptive statistics for self-reported education level AA: Associate of Arts.

Highest Education Level	Frequency	Percent
High school	38	28.36
Some college, no degree	18	13.43
AA/trade school	8	5.97
College	35	26.12
Graduate	25	18.66
Professional degree	7	5.22
Other	1	0.75
Not reported	2	1.49

Emotional scales

The mean PHQ-4 score for the cohort was 3.18 ± 3.18, indicating mild psychological distress. Of the anxiety and depression subscales, 41 (30.6%) and 32 (23.9%) of the population were positive, respectively. Furthermore, anxiety/depression subscales were analyzed in combination with one another for positive or negative screenings. Anxiety and depression screenings were positive/positive for 21 (15.7%) participants, 20 (14.9%) were positive/negative, 11 (8.2%) were negative/positive, and 82 (61.2%) were negative/negative, respectively. 

**Figure 1:**
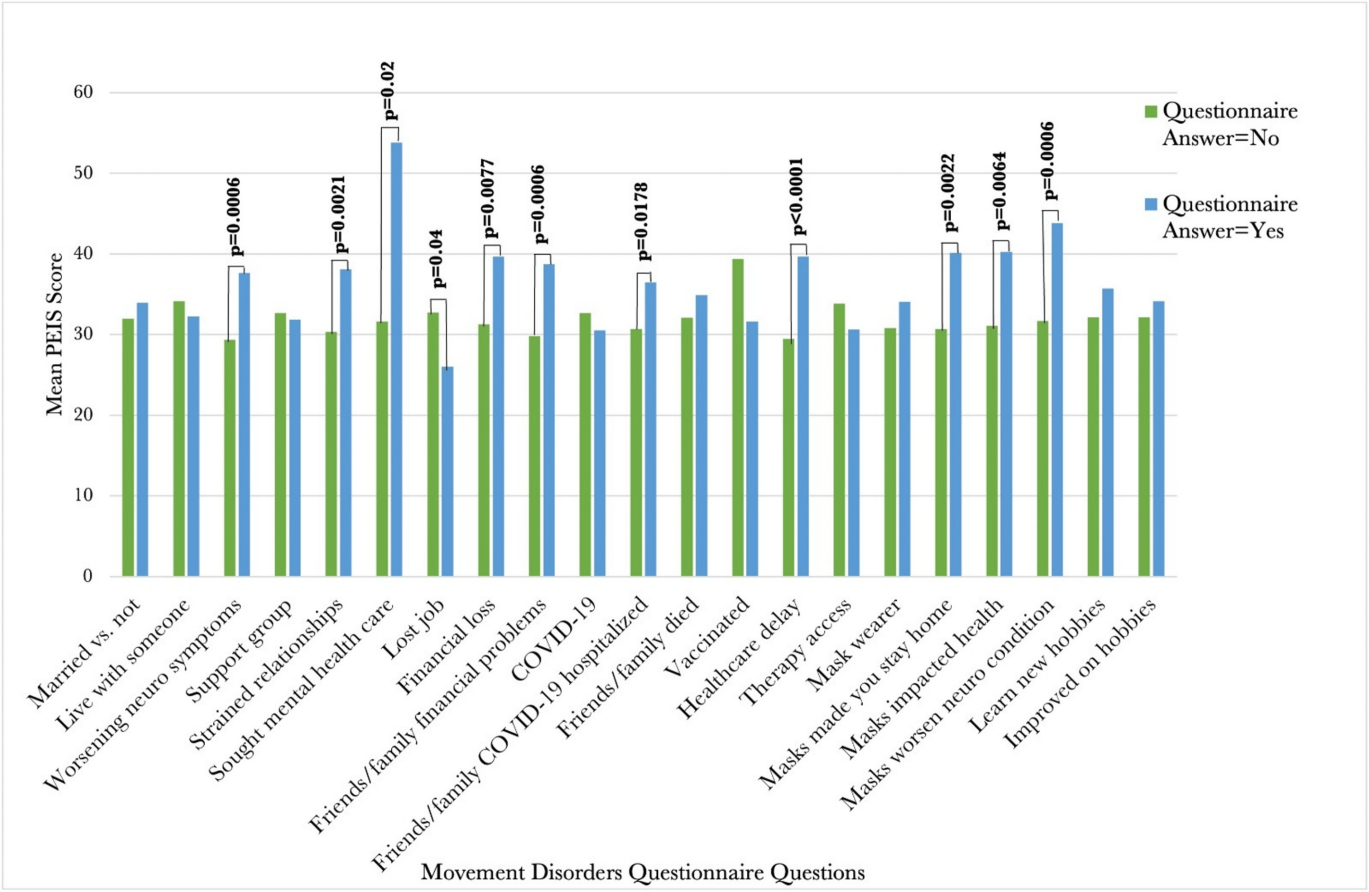
A comparison of mean PEIS scores for varying questionnaire topics. Mean PEIS scores were calculated, and associations between these variables and clinical rating scale responses were analyzed using the independent t-test. PEIS: Pandemic Emotional Impact Scale; COVID-19: coronavirus disease 2019.

The mean PEIS score for the cohort was 32.52 ± 12.61 (range 16-80), compared to a mean score of 25.37 reported by Ballou and colleagues in individuals who have not experienced COVID-19 in their household [11]. When analyzing PEIS scores and answers from the questionnaire, it was found that patients who answered the question “yes” had higher average PEIS scores for the following questions: “worsening neuro symptoms” (p=0.0006), “strained relationships” (p=0.0021), “sought mental health care” (p=0.02), “financial loss” (p=0.0077), “friends/family financial loss” (p=0.0006), “friends/family hospitalized from COVID-19” (p=0.0178), “healthcare delay” (p<0.0001), “masks made you stay home” (p=0.0022), “masks impacted health” (p=0.0064), and “masks worsened neuro condition” (p=0.0006) (Figure 1). The top three prompts that recorded the highest score were “Feeling more frustrated about not being able to do what you usually enjoy doing," “More worried about the health and safety of family members or friends," and “More worried about your personal health and safety." The three prompts that recorded the lowest score were “More worried about getting necessities like groceries or medications," “Having more difficulty concentrating," and “Feeling more angry or irritated."

**Figure 2:**
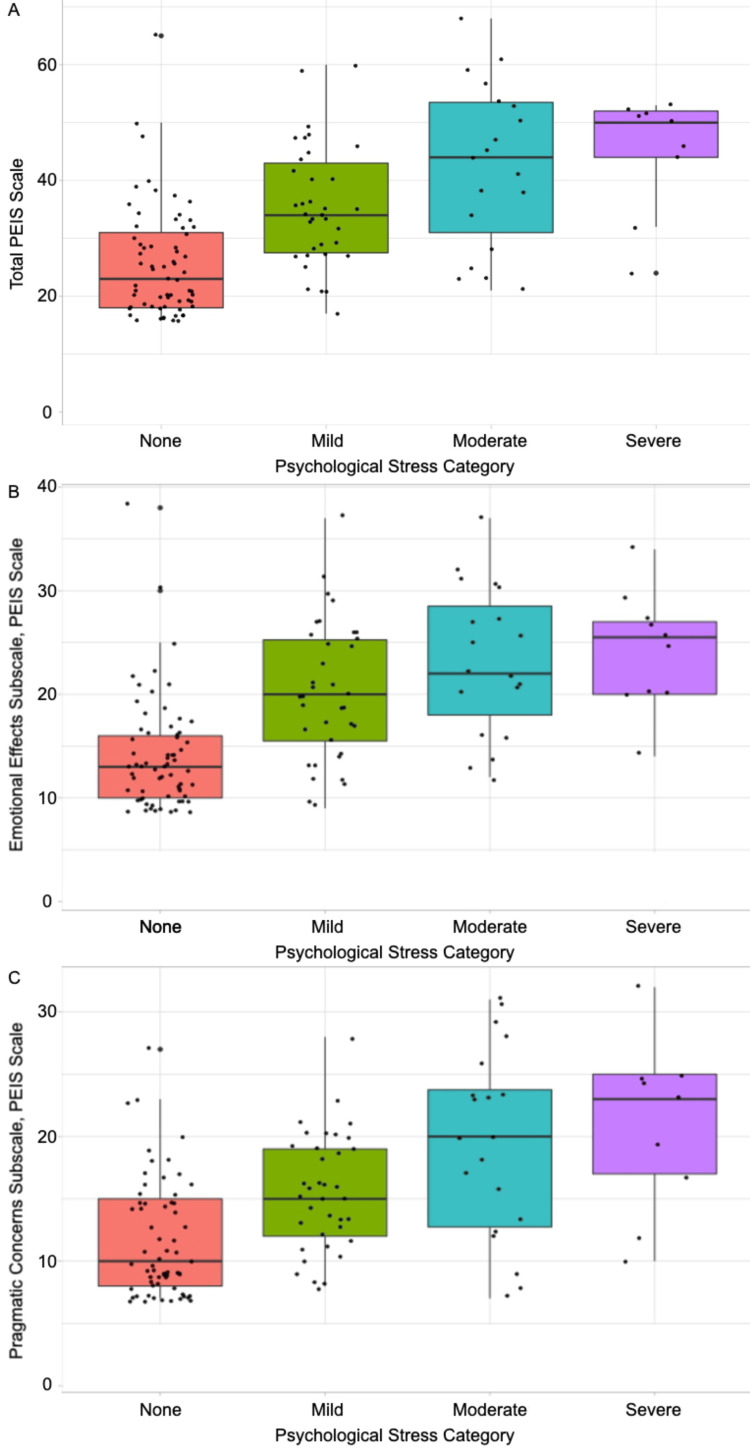
Comparison of PHQ-4 severity with PEIS score (A) The PEIS total score was represented based on the psychological stress category. (B) The PEIS emotional effects subscale score was represented based on the psychological stress category. (C) The PEIS pragmatic concerns subscale score was represented based on the psychological stress category. PEIS: Pandemic Emotional Impact Scale.

To further elucidate the association between the PHQ-4 and PEIS severity, we ran several Jonckheere-Terpstra tests on both the overall scales and subscales. There was a significant association between the PEIS total score and the severity of the PHQ-4 (p<0.0001) (Figure 2A). Thus, if the patient reported a higher PEIS score, then they were more likely to be categorized in a more severe psychological distress classification. Furthermore, there was a significant association between the PEIS subscales (emotional and pragmatic) and the severity of the PHQ-4 (p<0.0001) (Figures 2B, 2C). In each of the two PEIS subscales, the same trend was demonstrated. If patients were to report a higher total score of either of the two PEIS subscales, their PHQ-4 classification would likely be more severe.

## Discussion

This study examined some important aspects of the emotional impact, stress, and burden of the COVID-19 pandemic on patients with movement disorders. Overall, we found that a significant number of patients with movement disorders participating in this study were under increased distress during the COVID-19 outbreak, as was the general population. The pandemic presented stressful situations for these patients in several diverse ways, given the delays in healthcare and worsening neurological symptoms potentially associated with reduced physical activity and pandemic-related social restrictions. Social factors, including financial concerns and strained relationships, were also amplified by the circumstances and likely contributed to a higher perception of stress and burden during the pandemic as well. Furthermore, a large population study based upon a Scottish primary care database described the increased number of physical and non-physical co-morbidities in patients with PD compared to those without PD, including coronary artery disease, cerebrovascular disease, and heart failure [9]. These are important risk factors that significantly increase the risk for more severe forms of COVID-19, suggesting that patients with movement disorders may be predisposed to worse outcomes [10].

In addition to assessing psychological distress, this study also explored the relationship between participants' experiences during the pandemic and their emotional well-being, as measured by the PEIS scale. It was found that patients who answered "yes" to questions about worsening neurological symptoms, strained relationships, seeking mental health care, financial loss, and healthcare delays had higher average PEIS scores. This may suggest that the pandemic's impact on individuals' lives and health has a significant association with their emotional well-being.

The PEIS scale has been validated in the general population, showing strong positive correlations with anxiety, depression, stress, and negative correlations with quality of life and happiness [11]. However, there are no studies to date assessing PEIS results in the context of patients with movement disorders. Further analysis of our data found a significant association between the PHQ-4 severity and the PEIS total score and the emotional and pragmatic subscales. These findings indicate that individuals who experienced more severe psychological distress also reported more negative impacts from the pandemic on their lives and health, and act to further validate the utility of the PEIS scale as a comprehensive measure of the multi-faceted psychological effects of the COVID-19 pandemic in the context of patients with movement disorders. These scales can be of great utility to neurologists and other healthcare providers to better understand the mental state of their patients with movement disorders and to provide an improved, personalized care regimen for each patient.

Our data revealed that an appreciable portion of the participants had clinically significant psychological distress, as shown using the PHQ-4 scale. The scale displayed a mean score of 3.18 ± 3.18, indicating mild psychological distress across the cohort, with positive results in 41 (30.6%) and 32 (23.9%) of the population on the anxiety and depression subscales, respectively. These results suggest the participants experienced some psychological distress, but the overall severity was mild. As compared to a large population-based study assessing PHQ-4 results in the German general population, levels of anxiety and depression were much higher in our cohort as compared to generalized data showing PHQ-4 scaled anxiety and depression prevalence to be only 289 (9.8%) and 307 (10.4%), respectively [13]. However, it is unclear whether these higher levels of depression and anxiety, as compared to the general population, are a direct consequence of the COVID-19 pandemic or are simply baseline levels for the population, given that the percentage of patients with movement disorders with pre-existing diagnoses of depression and anxiety are estimated to be between 20-30% and 20-52%, respectively [14]. Nonetheless, there is evidence to suggest that stress in general is a significant factor to consider in movement disorder symptoms, as it is known to exacerbate symptoms such as tremor [15], freezing gait [16], and dyskinesia [17], particularly in PD [18]. Furthermore, stress has also been shown to worsen nonmotor (pain perception, constipation, etc.) symptoms of PD [19] as well. Finally, cognitive stress has also been seen to reduce levodopa efficacy in PD patients [20]. Although the exact mechanism of stress and its ability to worsen PD symptoms is not yet clear, it is suspected that the dysfunction of dopaminergic neurons may make patients more susceptible to stress [18]. These results emphasize the importance of psychological screenings in patients with movement disorders, specifically in the context of ongoing epidemic emergencies. Furthermore, the results may help inform strategies to support patients with movement disorders during future public health emergencies, although the development of formal care frameworks warrants further study.

While the presented results provide valuable insights, some limitations should be considered when interpreting them. For example, the study's cross-sectional design and lack of pre-pandemic baselines limit the ability to establish causality between participants' experiences during the pandemic and their emotional well-being. Additional variables that may contribute to emotional well-being, such as comorbid neurological conditions and medications, were not collected. It is worth noting that the majority of the participants were Caucasian (91%), and all could speak and read English, which could limit the generalizability of the findings to more diverse populations. Additionally, the study's focus on a relatively homogeneous population may limit generalizability to other groups. Future research could expand the sample size, include a more diverse population to further explore these relationships, and assess the potential long-term effects of COVID-19 on patients with movement disorders. Although it has been established that the prevalence and incidence of movement disorders such as PD and ET are higher in men than in women, the relationship between race and PD has been controversial, with many studies finding contradictory findings [21,22]. More recently, a study done by Dahodwala et al. showed that there is a lower diagnosed incidence of PD among African Americans than Caucasians, suggesting that PD is being under-recognized in African Americans, potentially as a result of racial disparities in healthcare [23,24]. Improved diagnostic rates across ethnic minorities will improve patient care and may elicit new trends or further support our results concerning the relationship between the pandemic’s effects on pragmatic and emotional outcomes.

## Conclusions

Overall, the presented results suggest that the COVID-19 pandemic was associated with changes in the emotional well-being of individuals with movement disorders, particularly those who endured negative experiences in their lives and health during this time. Patients with movement disorders experienced elevated levels of anxiety and depression, most notably pandemic-related stressors, including healthcare delays, worsening neurological symptoms, financial challenges, and increased psychological stress. These findings emphasize the vulnerability of this population during global crises and have important implications for healthcare providers and policymakers, highlighting opportunities to strengthen clinical care delivery, including integrating telehealth visits to maintain continuity of neurological care, expanding access to remote physical and occupational therapy, and incorporating routine psychosocial screening to identify patients at risk for distress. Future research should aim to expand these findings to more diverse populations, evaluate long-term neuropsychiatric and functional outcomes, and identify targeted interventions that mitigate psychosocial stressors in patients with movement disorders during public health emergencies.

## Appendices

**Table 4:** COVID-19 and movement disorders questionnaire

Identifier:	
Date:	
Demographics	
1	Age:
2	Gender:
3	Are you of Hispanic or Latino descent:
	Yes
	No
4	Race (please select one or more)
	White
	Asian
	American Indian or Alaska Native
	Black or African American
	Native Hawaiian or other Pacific Islander
5	What is your neurological diagnosis?
5a	Year of diagnosis:
6	What is your highest level of education?
7	Employment status:
8	Marital status:
9	Do you live with a spouse, partner, or caregiver?
10	Do you smoke or have you ever smoked?
COVID-19 Impact	
11	Do you believe your neurological symptoms have worsened during the pandemic? If yes, please explain.
12	Do you attend a support group for your neurological illness?
13	If you have experienced stress during the pandemic, what was the major cause?
14	Did the pandemic cause you to seek mental health care?
15	Did you lose your job due to COVID-19?
15a	If so, why?
16	Did you suffer financial loss due to COVID-19?
16a	If so, why?
17	Did any of your friends or family undergo financial hardship due to the pandemic?
18	Were you ever diagnosed with COVID-19?
18a	If so, were you hospitalized?
19	Were any of your friends or family hospitalized with COVID -19?
20	Did any of your friends or family die from COVID-19?
21	Have you been vaccinated for COVID -19?
22	Do you want to be vaccinated for COVID-19?
22a	If no, what are your reason(s) for not wanting to get vaccinated?
23	Where do you get most of your information about COVID-19?
24	Was your healthcare delayed or unavailable due to COVID-19 at any time?
25	Have you had any doctor appointments by telehealth?
26	Were you satisfied with your telehealth visits?
26a	If not, please elaborate the reason(s):
27	Do you want to use telehealth in the future?
28	Do you wear a mask at all times when outside your home?
29	What kind(s) of mask(s) do you use?
30	How often do you change your mask?
31	Where have you obtained your masks?
32	How much money do you estimate you have spent buying masks during the COVID-19 pandemic?
33	What is the worst part of wearing a mask?
34	Has wearing a mask caused you to stay home rather than go outdoors due to discomfort?
35	35. Has wearing a mask impacted your health?
35a	If so, how? (Such as ear pain, head pain, sore throat, difficulty breathing, sinus issues, strap pain, teeth issues, speech, eye pressure, etc.).
36	Does wearing a mask worsen your neurological condition?
36a	If so, how?
